# Developing risk models and subtypes of autophagy-associated LncRNAs for enhanced prognostic prediction and precision in therapeutic approaches for liver cancer patients

**DOI:** 10.32604/or.2023.030988

**Published:** 2024-03-20

**Authors:** LU ZHANG, JINGUO CHU, YUSHAN YU

**Affiliations:** Department of General Practice, The First Affiliated Hospital of Ningbo University, Ningbo, China

**Keywords:** Autophagy, Hepatocellular carcinoma (HCC), Prognosis, Precision medicine, Paclitaxel

## Abstract

**Background:**

Limited research has been conducted on the influence of autophagy-associated long non-coding RNAs (ARLncRNAs) on the prognosis of hepatocellular carcinoma (HCC).

**Methods:**

We analyzed 371 HCC samples from TCGA, identifying expression networks of ARLncRNAs using autophagy-related genes. Screening for prognostically relevant ARLncRNAs involved univariate Cox regression, Lasso regression, and multivariate Cox regression. A Nomogram was further employed to assess the reliability of Riskscore, calculated from the signatures of screened ARLncRNAs, in predicting outcomes. Additionally, we compared drug sensitivities in patient groups with differing risk levels and investigated potential biological pathways through enrichment analysis, using consensus clustering to identify subgroups related to ARLncRNAs.

**Results:**

The screening process identified 27 ARLncRNAs, with 13 being associated with HCC prognosis. Consequently, a set of signatures comprising 8 ARLncRNAs was successfully constructed as independent prognostic factors for HCC. Patients in the high-risk group showed very poor prognoses in most clinical categories. The Riskscore was closely related to immune cell scores, such as macrophages, and the DEGs between different groups were implicated in metabolism, cell cycle, and mitotic processes. Notably, high-risk group patients demonstrated a significantly lower IC50 for Paclitaxel, suggesting that Paclitaxel could be an ideal treatment for those at elevated risk for HCC. We further identified C2 as the Paclitaxel subtype, where patients exhibited higher Riskscores, reduced survival rates, and more severe clinical progression.

**Conclusion:**

The 8 signatures based on ARLncRNAs present novel targets for prognostic prediction in HCC. The drug candidate Paclitaxel may effectively treat HCC by impacting ARLncRNAs expression. With the identification of ARLncRNAs-related isoforms, these results provide valuable insights for clinical exploration of autophagy mechanisms in HCC pathogenesis and offer potential avenues for precision medicine.

## Introduction

Hepatocellular carcinoma (HCC), a common gastrointestinal tumour, is the second primary cause of death from the disease [1]. According to statistics, over 500,000 new patients suffer from HCC every year, and the annual death rate is over 700,000 [2]. HCC is one frequently seen malignant tumor, and many factors have been verified to be implicated in its development, such as liver cirrhosis, alcoholic liver disease, viral infection, diabetes mellitus as well as a nonalcoholic fatty liver disease [3–5]. At the current stage, the effective treatments options for HCC mainly include percutaneous approach treatment, liver transplantation and hepatectom [6]. However, many individuals will experience recurrence or distant metastases following surger [7]. Treatment can only give minimal therapeutic advantages for more than 70% of HCC patients in the advanced stage [8]. Accordingly, it is urgent to search for a novel and reliable screening method to improve the diagnostic accuracy and therapeutic effect to enhance the prognosis. Precision medicine has the potential to better serve the heterogeneity of individuals. If a new and reliable screening method can be found to screen high-risk populations, diagnostic accuracy can be improved, more targeted treatments can be provided, medical resources can be saved, and patient outcomes can be improved [9,10]. It is important to seek out such methods to maximize the benefits of precision medicine.

Autophagy, as a catabolic process, involves the multi-step degradation of protein and organelles. It participates a part in sustaining cell homeostasis, which is associated with heart disease, ageing, neurodegeneration as well as cancer development [11]. According to prior research, autophagy takes multiple play a role in the occurrence, maintenance as well as development of tumour [12]. According to genetic evidence, as a mechanism of tumour inhibition, autophagy can contribute to the survival of tumours under chemotherapy and stress [13]. Additionally, a growing number of data sugguest that autophagy in cancer cells is regulated by long-chain non-coding RNA (lncRNA) [14]. With a length of over 200 bp [15], lncRNA has been verified to have extensive impacts on a variety of crucial biological processes, like cell differentiation, proliferation, RNA attenuation, RNA splicing, genetic regulation of gene expression, protein folding as well as microRNA (miRNA) regulation [16–19]. Reportedly, lncRNA HULC induces liver cancer by suppressing PTEN through autophagy cooperation with miR15a [20,21]. However, these studies focus on single or few lncRNA associated with HCC. There is no lncRNA expression profile in the TCGA database is available to explore new biomarkers for predicting HCC prognosis. In addition, it is still unknown whether these molecular targets or their constructed models are relevant to the response of immunotherapy, leaving room for breakthroughs to be sought after. One of the major challenges in driving clinical treatment for HCC is identifying high-risk patients at an early stage and using more targeted drugs in combination with immunotherapy to enhance drug response, reduce drug resistance, and provide more options for second-line complementary therapies [22,23]. It is crucial to address these challenges in research on HCC clinical treatment.

Retrieval of 232 Autophagy Genes from the HADb Database to Explore ARLncRNAs Expression Profiles, and evaluating Risk Models for Predictive Value and Targeting Sensitive Medications in High-Risk HCC Patients for Prognostic Prediction and Clinical Drug Guidance.

## Materials and Methods

### Data gathering and processing

RNA sequencing (RNA-TPM) and clinical data concerning HCC patients were sourced from the TCGA (The Cancer Genome Atlas) Genomic Data Commons (GDC) portal (https://portal.gdc.cancer.gov/repository). Following the exclusion of samples with incomplete clinical information, a collection of 373 tumor samples, along with corresponding paracancerous normal samples, was obtained. Autophagy gene data were accessed from the Human Autophagy Database (HADb) (http://www.autophagy.lu/index.html), consisting of 232 entries.

### Prognostic signature development and verification

ARLncRNAs linked to autophagy-related genes were identified through Pearson analysis, using R > 0.4 and *p* < 0.04 as the selection criteria. The expression matrix of ARLncRNAs was then combined with survival data, and a univariate Cox regression analysis was conducted. This determined a significant correlation between autophagy-related lncRNAs and overall survival (OS), considering *p* < 0.05 as statistically significant, using the “Survival” R package.

Following this, LASSO regression analysis was performed, leading to the selection of specific autophagy-related lncRNAs. These were further subjected to multivariate Cox regression analysis, and through the application of forward and backward selection algorithms, the most suitable model was obtained. The weighted regression coefficients from this analysis were used to characterize prognostic features.

A risk score for each patient was calculated using the following formula: Riskscore = (X: coefficients for each gene, Y: expression of each gene). Utilizing the “Surv Miner” R package, the samples were categorized into high or low-risk groups based on the median risk score.

The “Survival” R software package was employed to plot Kaplan-Meier (K-M) survival curves, assessing the survival difference between the two groups. The model’s accuracy was evaluated using the area under the curve (AUC) and the consistency index (C-index) of the dynamic ROC curve over time. This same formula was applied to verify the stability of the prognostic model in the test group.

### Nomogram construction

The R package “RMS” was used for constructing a nomogram for evaluating the patients’ 1-, 3- and 5-year survival rates. Subsequently, the calibration curve of the nomogram was generated, followed by consistency evaluation between the actual observed value and predicted value via the rms package.

### Immunological analysis

The immunity scores of the samples were computed utilizing the CIBERSORT and MCP-counter algorithms. The relationship between these immunity scores and the Riskscore was subsequently analyzed using Spearman correlation. Butterfly plots were crafted to represent the correlation network, leveraging the ggClusterNet package, and scatter plots were drawn to visualize the association between Riskscore and various immune cells, facilitated by the ggstatsplot package. Additionally, the Tumor Immune Dysfunction and Exclusion (TIDE) algorithm was employed to forecast potential immunotherapy responses across the samples, further enhancing the study’s depth of understanding in the immune landscape of the disease. The OCLR algorithm was employed to determine the Stem Score of the tumors. This score was then mapped to the [0,1] range through a linear transformation that involved subtracting the minimum value and dividing by the maximum. Subsequent to this transformation, an analysis was conducted to explore the correlation between gene expression and the Stem Score, utilizing Spearman correlation as the method of assessment. This approach provided insights into the relationship between stemness characteristics and the underlying genetic factors within the tumors.

### Drug sensitivity prediction

To identify potential anti-HCC drugs and evaluate their clinical applications, the R packages “pRRophetic” and “oncoPredict” were utilized to predict the half-maximal inhibitory concentration (IC50) of various chemotherapy drugs based on the Cancer Genome Project (CGP) database and Cancer Drug Sensitivity Genomics (GDSC) database. Drugs with sensitivity differences between different groups were identified.

### Cell culture and qRT-PCR

The human hepatic astrocytes cell line LX-2 and the human HCC cell line HepG2 were purchased from Zhejiang Mason Cell Technology Co. (Hangzhou, China). Cell culture conditions and qRT-PCR were as described in previous studies [24]. The list of primer sequences is described in Suppl. Table 1.

### Functional analysis

Differentially expressed genes (DEGs) between low and high-risk groups were identified using the “limma” package, with selection criteria set at an adjusted *p*-value of <0.05 and an absolute log2fold change (FC) of ≥0.5. Subsequent analyses were performed to understand the underlying biological functions among the different groups. Specifically, the “limma” and “clusterProfiler” packages were utilized to conduct gene ontology (GO) analysis and Kyoto Encyclopedia of Genes and Genomes (KEGG) pathway enrichment analysis. Additionally, Gene Set Enrichment Analysis (GSEA) was employed, providing a comprehensive exploration of the biological pathways and functions that distinguish the risk groups.

### Construction of autophagy-related HCC subtypes

The expression profiles were subjected to consistency analysis using the ConsensusClusterPlus package, setting a maximum of six clusters. By employing 100 repetitions, 80% of the total samples were extracted, utilizing the parameters clusterAlg = “hc” and innerLinkage = ‘ward.D2’. Clustering heatmaps were generated using the R package, pheatmap. Within the gene expression heatmaps, only genes with a variance greater than 0.1 were retained. Should the number of input target genes exceed 1000, the top 25% of genes were extracted for display, following a sorting process that arranged them according to variance value, from high to low.

## Results

### Screening of prognostic genes of lncRNA

**Figure 1:**
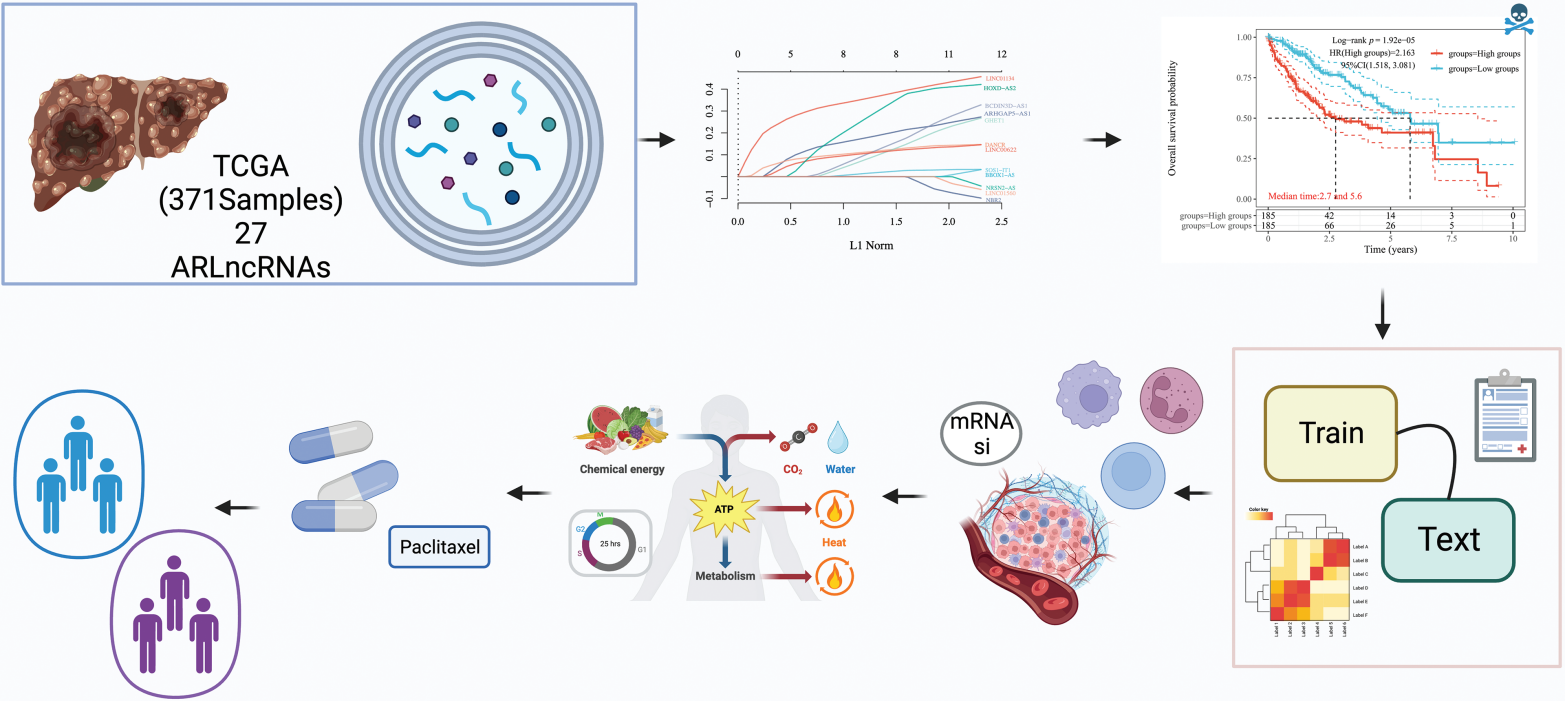
Study workflow diagram. The objective of this study was to explore the autophagy-related long non-coding RNAs (ARLncRNAs) signatures, aiming to predict the survival rates of hepatocellular carcinoma (HCC) patients. Utilizing The Cancer Genome Atlas (TCGA) database, a total of 371 HCC samples containing lncRNA expression profiles were subject to a rigorous analysis. This analysis included univariate Cox regression, Lasso regression, and multivariate Cox regression techniques to pinpoint risk models for prognostically significant lncRNA constructs. The process also encompassed validation of the models’ validity, comparisons of immune function variations between different risk groups, examination of possible biological pathways, and screening for optimal pharmaceutical interventions for high-risk patients. Additionally, the study sought to classify autophagy-related subtypes, further enriching our understanding of HCC.

**Figure 2:**
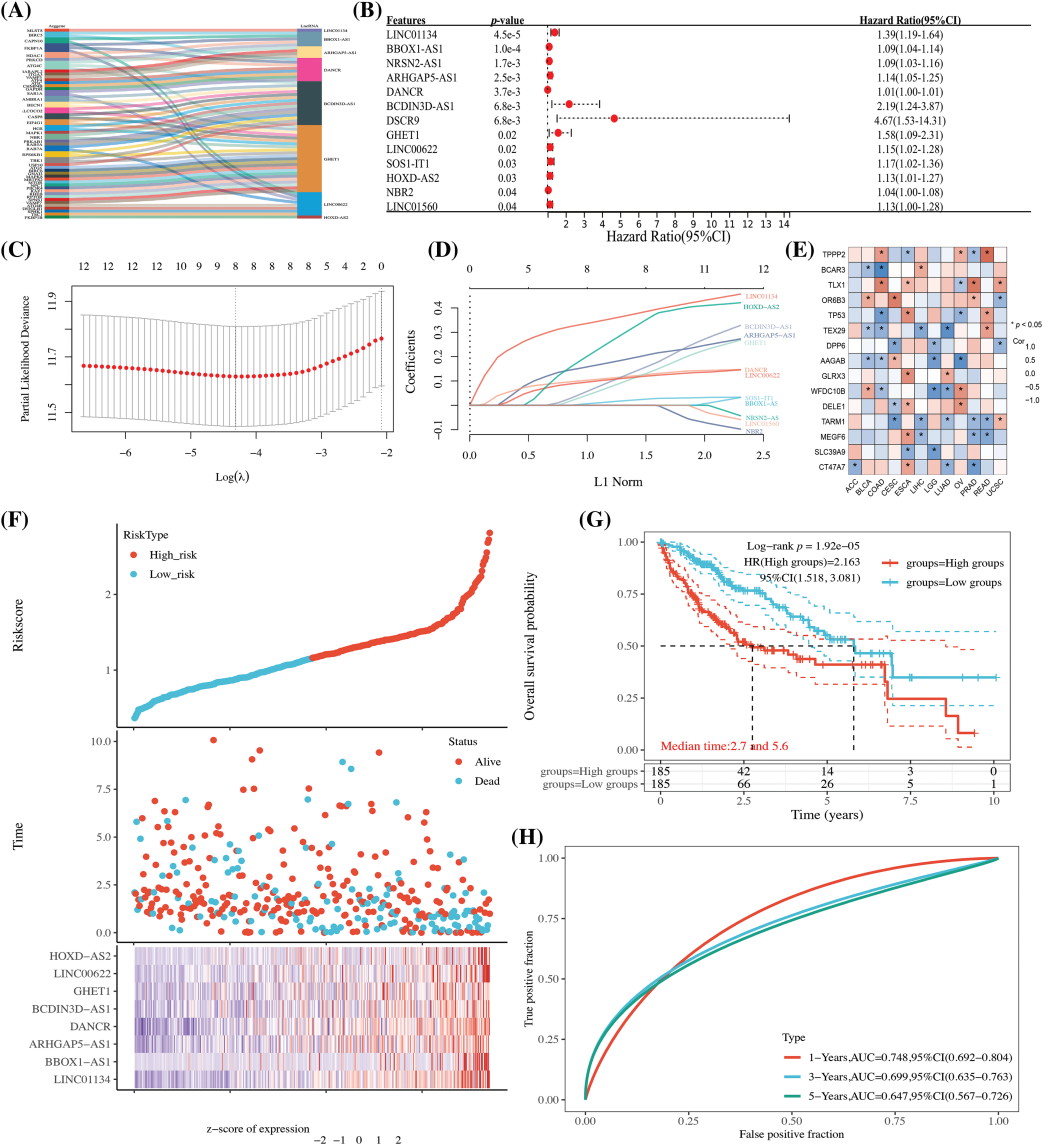
The establishment of an HCC autophagy-related lncRNA prognostic model. (A) A Sankey diagram displaying lncRNAs linked to autophagy genes; (B) One-way Cox analysis of autophagy-related lncRNAs (ARLncRNAs) and their association with HCC prognosis; (C) Coefficient selection following Lasso methodology; (D) A variable trajectory diagram; (E) A heatmap representing the correlation between the eight ARLncRNAs and autophagy genes; (F) A risk factor analysis comparing high- and low-risk groups; (G) Kaplan-Meier (K-M) curves to depict survival rates for both high and low-risk groups; (H) Time-dependent receiver operating characteristic (TPR) curves to assess the model’s predictive accuracy. Note: *p < 0.5.

In Fig. 1, the core design process of this study is illustrated, resulting in the identification of 27 lncRNAs that are associated with autophagy genes, as determined through the expression profiling of autophagy-related genes (refer to Fig. 2A and Suppl. Table 2). Additionally, 13 lncRNAs, which are linked to HCC prognosis, were discerned through screening via univariate Cox analysis (see Fig. 2B). These findings will be pivotal for subsequent investigations.

### Establishment of prognosis model of HCC autophagy-associated lncRNAs

Utilizing the 13 identified autophagy-related prognostic lncRNAs, we conducted a LASSO Cox analysis to build a prognostic model for autophagy-related lncRNAs. Specifically, the Riskscore was formulated as follows: Riskscore = (0.393) * LINC01134 + (0.0278) * BBOX1-AS1 + (0.2136) * ARHGAP5-AS1 + (0.1299) * DANCR + (0.1731) * BCDIN3D-AS1 + (0.1451) * GHET1 + (0.123) * LINC00622 + (0.3735) * HOXD-AS2. This equation embodies the mathematical relationship uncovered through our analysis, serving as a tool to predict the overall survival (OS) prognosis for patients diagnosed with HCC. As a consequence, eight lncRNA signatures were identified and screened, showing a strong correlation with autophagy genes (see Fig. 2E). Patients were then categorized into high and low-risk groups based on the median value. Those in the high-risk group were found to have a less favorable prognosis and reduced survival rates (as depicted in Figs. 2F and 2G). Time-dependent curves further corroborated the model’s value in evaluation and analysis (refer to Fig. 2H).

### A risk model comprising ARLncRNAs can be an independent risk factor for predicting HCC prognosis

**Figure 3:**
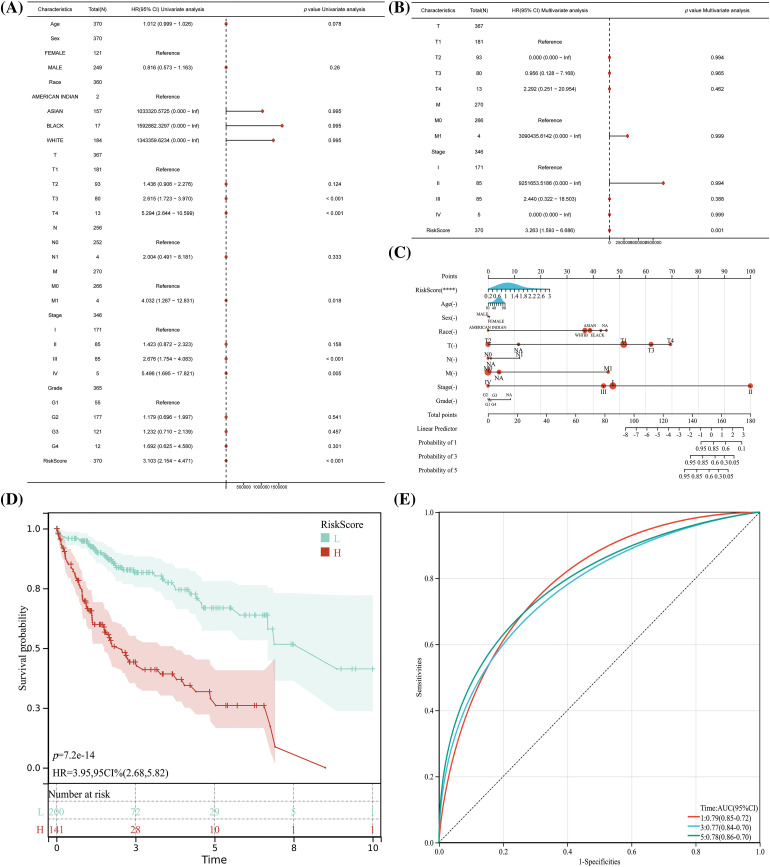
The risk model which comprised of ARLncRNAs, can act as an independent risk factor in predicting the prognosis for HCC patients. (A) Unifactorial and (B) Multifactorial regression analyses to evaluate the significance of Riskscore along with other clinical characteristics in determining HCC prognosis; (C) The creation of a nomogram for assessing the importance of Riskscore and accompanying clinical features in forecasting HCC prognosis; (D) K-M curves and (E) TPR curves to validate and confirm the Nomogram results. Note: ****p < 0.0001.

In the Cox analysis, other clinical confounders such as age and gender were incorporated. Subsequent univariate and multivariate Cox regression analyses revealed that the Riskscore, formed by ARLncRNAs, emerged as an independent risk factor affecting the prognosis of HCC patients (see Figs. 3A and 3B, *p* < 0.001). Additionally, we crafted a Nomogram column line table, reinforcing that the Riskscore is an unfavorable factor influencing the prognosis of HCC patients (refer to Fig. 3C, *p* < 0.001). Patients in the high-risk group continued to show a poorer prognosis (Fig. 3D, *p* < 0.001), and the model exhibited a definite level of precision (Fig. 3E, AUC > 0.7, *p* < 0.05).

**Figure 4:**
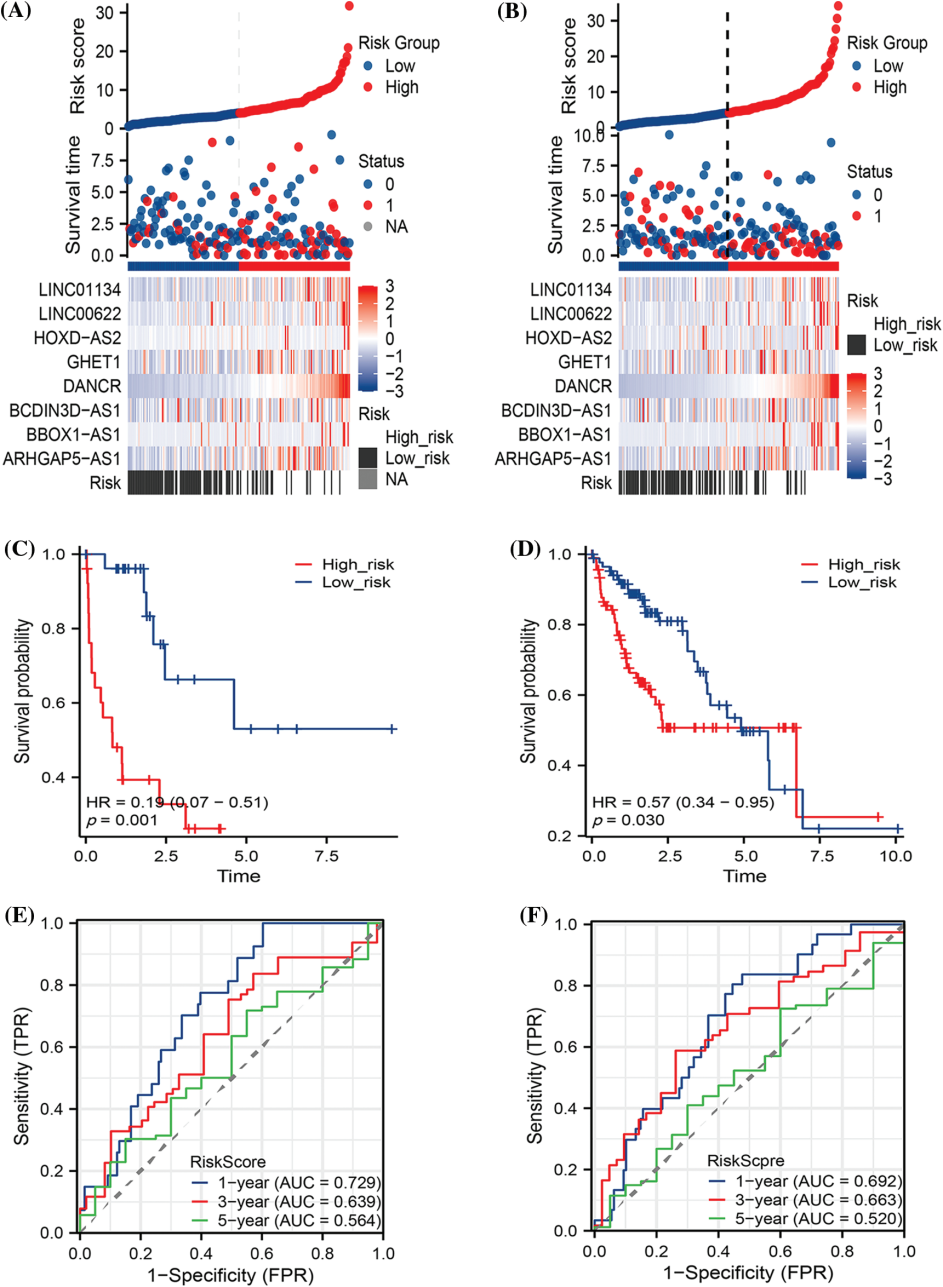
The prognostic predictions of the models within both the Train and Test sets. The diagram includes (A) Risk factor analysis for the Train set and (B) Test set; (C) Kaplan-Meier (K-M) curves pertaining to the Train set and (D) Test set; and (E) True Positive Rate (TPR) analysis for both the Train and (F) Test sets.

The HCC expression profiles were randomly divided into training and test sets based on a 1:1 ratio. In both the TRAIN SET and TEST SET, we discerned a consistent pattern where patients in the high-risk group faced a less favorable prognosis. This manifested as significantly reduced survival times (as shown in Figs. 4A and 4B) and lower survival rates (illustrated in Figs. 4C and 4D). Additionally, TPR exhibited a certain degree of predictive accuracy across both datasets, further substantiating our findings (refer to Figs. 4E and 4F).

**Figure 5:**
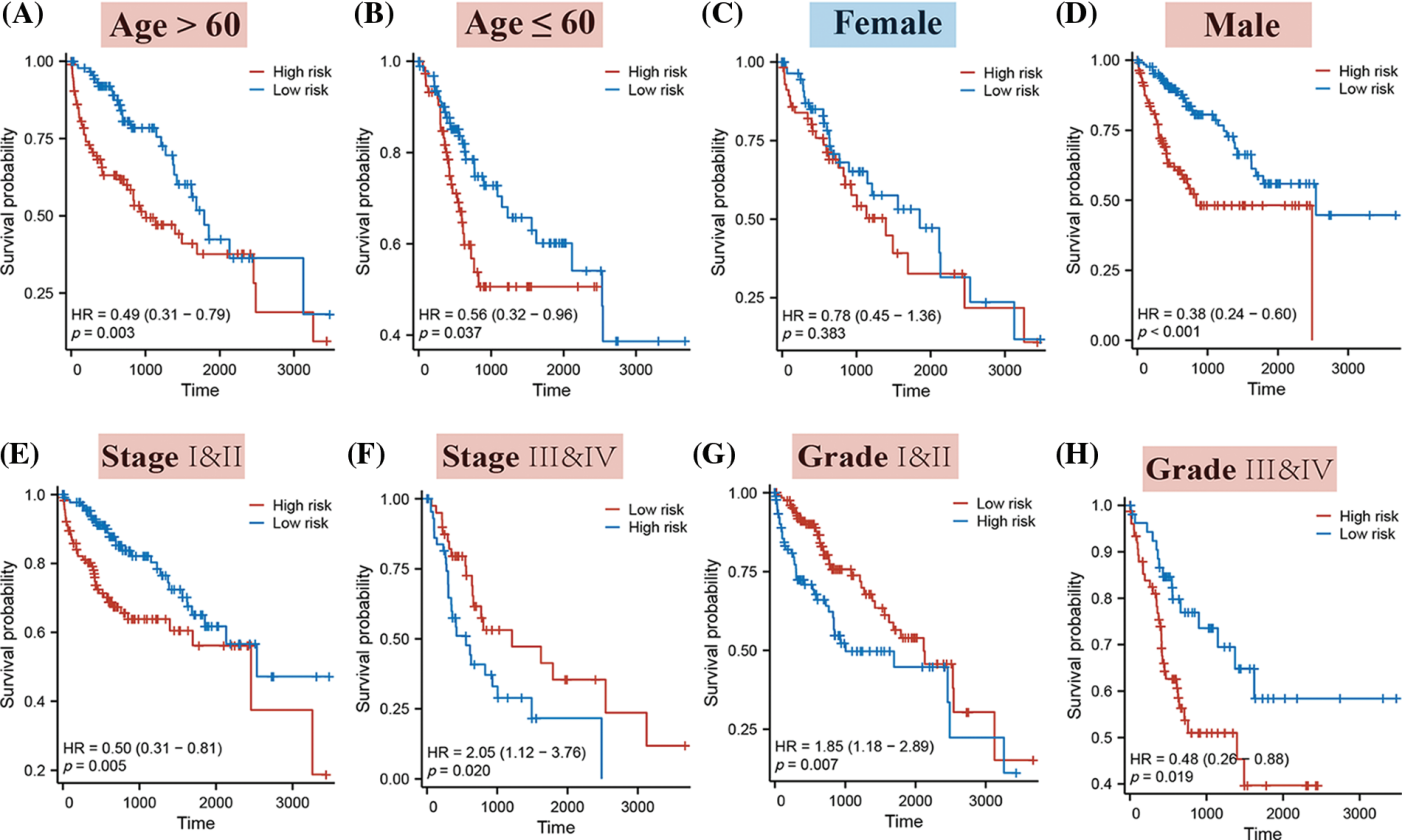
The K-M curves for high and low-risk patients, segmented by different clinical characteristics. Specifically, the K-M curves represent the following categories: (A) Patients aged over 60; (B) Patients aged 60 or younger; (C) Female patients; (D) Male patients; (E) Stages I & II; (F) Stages III & IV; (G) Grades I & II; (H) Grades III & IV.

The stratification of HCC patients based on various clinical characteristics demonstrated that a higher Riskscore typically corresponded to a decreased probability of occurrence in patients across all age groups, in males, and across all clinical stages and grades. Notably, this pattern was not observed in female patients (as illustrated in Fig. 5).

### Immune function and cancer progression in different risk groups

**Figure 6:**
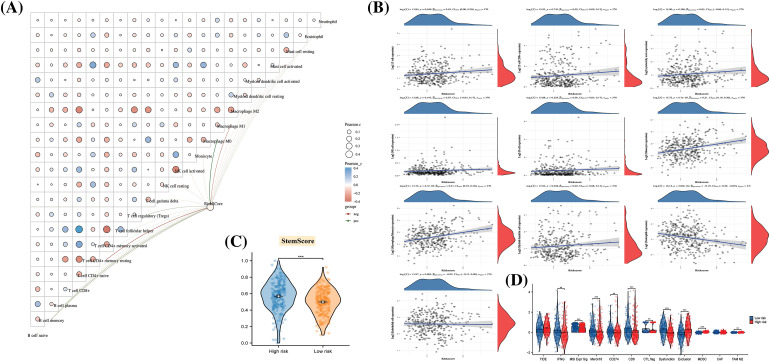
The disparities in immune function and cancer progression across different risk groups. (A) A butterfly plot reveals the correlation between Immune Score and Riskscore, as determined by the CIBERSORT algorithm; (B) The relationship between Immune Score and Riskscore, calculated using the MCP-counter algorithm; (C) Inter-sample predictions of potential immunotherapy response utilizing the Tumor Immune Dysfunction and Exclusion (TIDE) algorithm; (D) A comparison detailing the differences in Stem Score between various groups.

As depicted in Fig. 6A, Riskscore exhibited a strong association with immune cells, including macrophage M0, CD4+ T cells, and others, with a predominantly positive correlation observed with macrophage expression (Fig. 6B). While the Tumor Immune Dysfunction and Exclusion (TIDE) did not show significant differences between risk groups, the expression of Microsatellite Instability (MSI), Merck18, CD8, Dysfunction, Exclusion, MDSC, and TAM M2 scores was notably lower in the high-risk group (Fig. 6C). This finding implies a potential impairment in immune function for high-risk patients.

Furthermore, cancer progression appeared to be linked to a gradual loss of differentiated phenotype and the acquisition of progenitor or stem cell-like characteristics. An analysis of the Stem Score for different risk groups revealed a significantly higher value in high-risk patients compared to those in the low-risk group (Fig. 6D). This observation suggests that the tissues of high-risk patients may have undergone changes at the molecular level, reflecting alterations in their biological properties.

### Functional differences between risk levels

**Figure 7:**
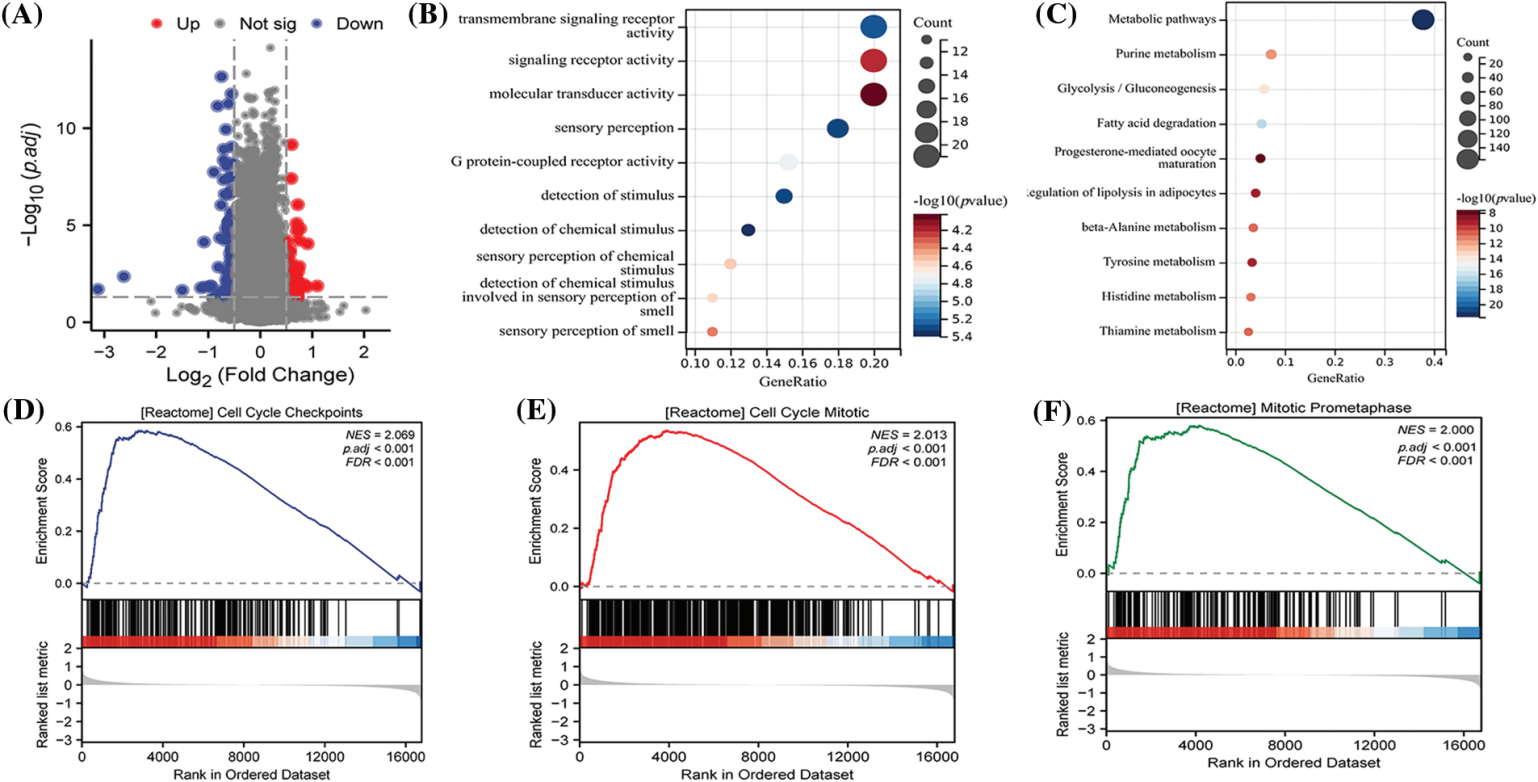
Biological pathways between different risk levels. (A) An analysis of differentially expressed genes (DEGs) between high and low-risk groups, conducted using the Limma package and visually represented as volcano plots; (B) GO (Gene Ontology) enrichment analysis to explore the potential biological functions of DEGs; (C) KEGG (Kyoto Encyclopedia of Genes and Genomes) enrichment to examine the signaling pathways that DEGs might be part of; Single Gene Set Enrichment Analysis (GSEA) results highlighting that DEGs are associated with (D) CELL CYCLE CHECKPOINTS; (E) CELL CYCLE MITOTIC; (F) MITOTIC PROMETAPHASE processes.

In order to investigate the underlying biological pathways distinguishing different risk groups, a comparison of differentially expressed genes (DEGs) was made between high and low-risk categories (Fig. 7A). Enrichment analysis revealed that these DEGs were predominantly concentrated in GO terms like transmembrane signaling receptor activity, G protein-coupled receptor activity, among others (Fig. 7C). These findings were further associated with terms such as Metabolic pathways and Fatty acid degradation (Fig. 7B), connecting them with specific signaling pathways (Fig. 7C). Additionally, GSEA results demonstrated that DEGs between different risk groups played a role in biological processes including cell cycle regulation and mitosis (Figs. 7D–7F).

### Differences in sensitivity to various drugs among patients were identified based on ARLncRNAs

**Figure 8:**
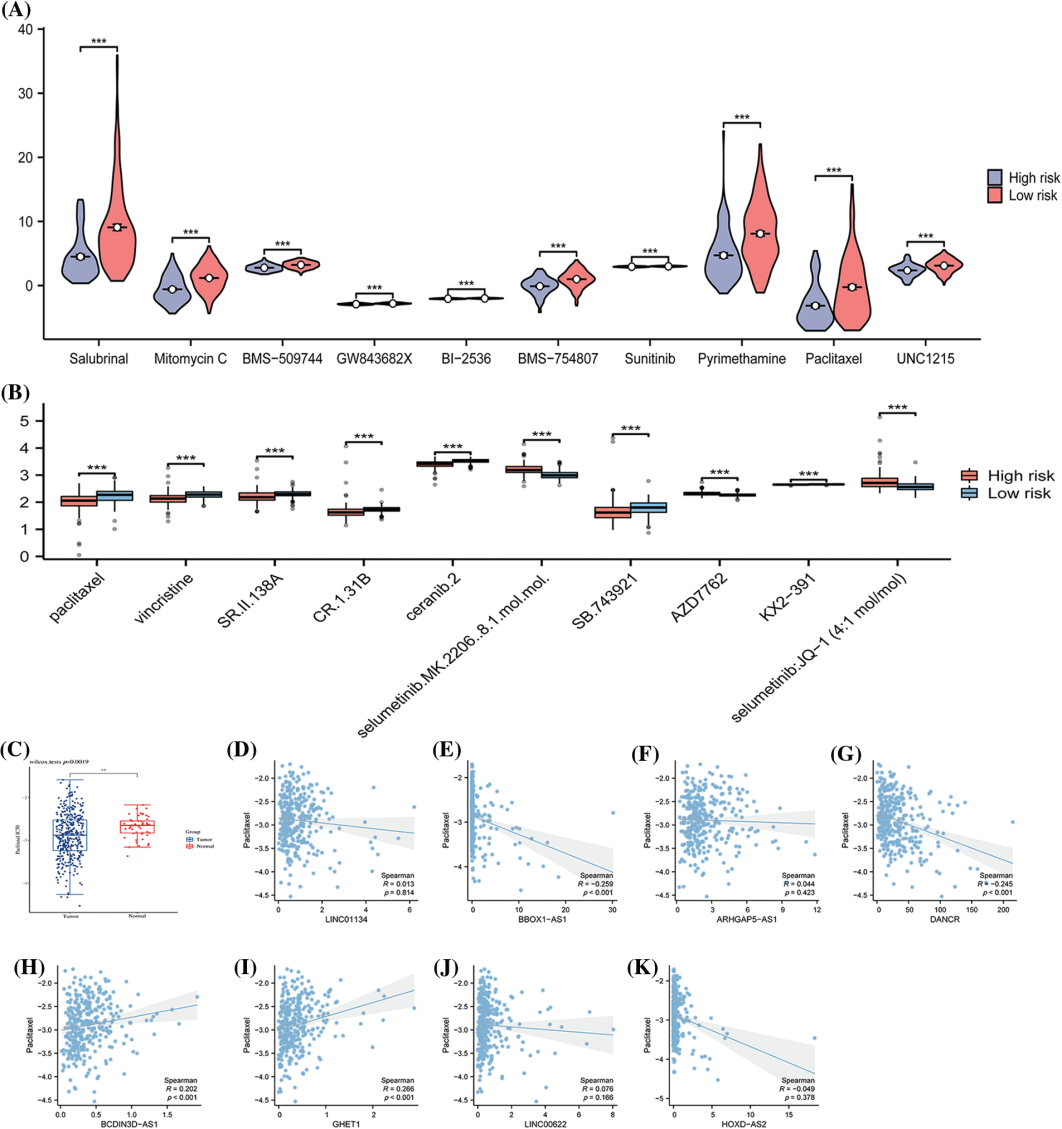
The variations in drug sensitivity among patients from different risk categories. The half-maximal inhibitory concentrations (IC50) of various chemotherapeutic drugs are calculated for samples of distinct risk classes using (A) the pRRophetic algorithm and (B) the oncoPredict algorithm. (C) Details a comparison of the IC50 of Paclitaxel between Tumor and Normal samples. The subsequent sections compare the IC50 with specific expressions: (D) LINC01134 and (E) BBOX1-AS1; (F) ARHGAP5-AS1; (G) DANCR; (H) BCDIN3D-AS1; (I) GHET1; (J) LINC00622; (K) HOXD-AS2. Note: *p < 0.5; *p < 0.01; ***p < 0.001; ****p < 0.0001.

Through the calculation of IC50 values in response to both conventional and chemotherapeutic drugs (as illustrated in Fig. 8), an analysis was conducted by examining the intersection of the top 10 drugs in the sensitivity rankings produced by two distinct algorithms (refer to Figs. 8A and 8B). This examination revealed a notable disparity in resistance to the drug Paclitaxel among various risk disease categories. Specifically, patients within the low-risk group exhibited significantly elevated IC50 values (as shown in Fig. 8C), a characteristic that might make them ideal candidates for treatment in the high-risk group. Further comparative analysis disclosed that the expression of BBOX1-AS1, DANCR, BCDIN3D-AS, and GHET1 in the samples held a significant.

### An examination of HCC subtypes based on ARLncRNAs

**Figure 9:**
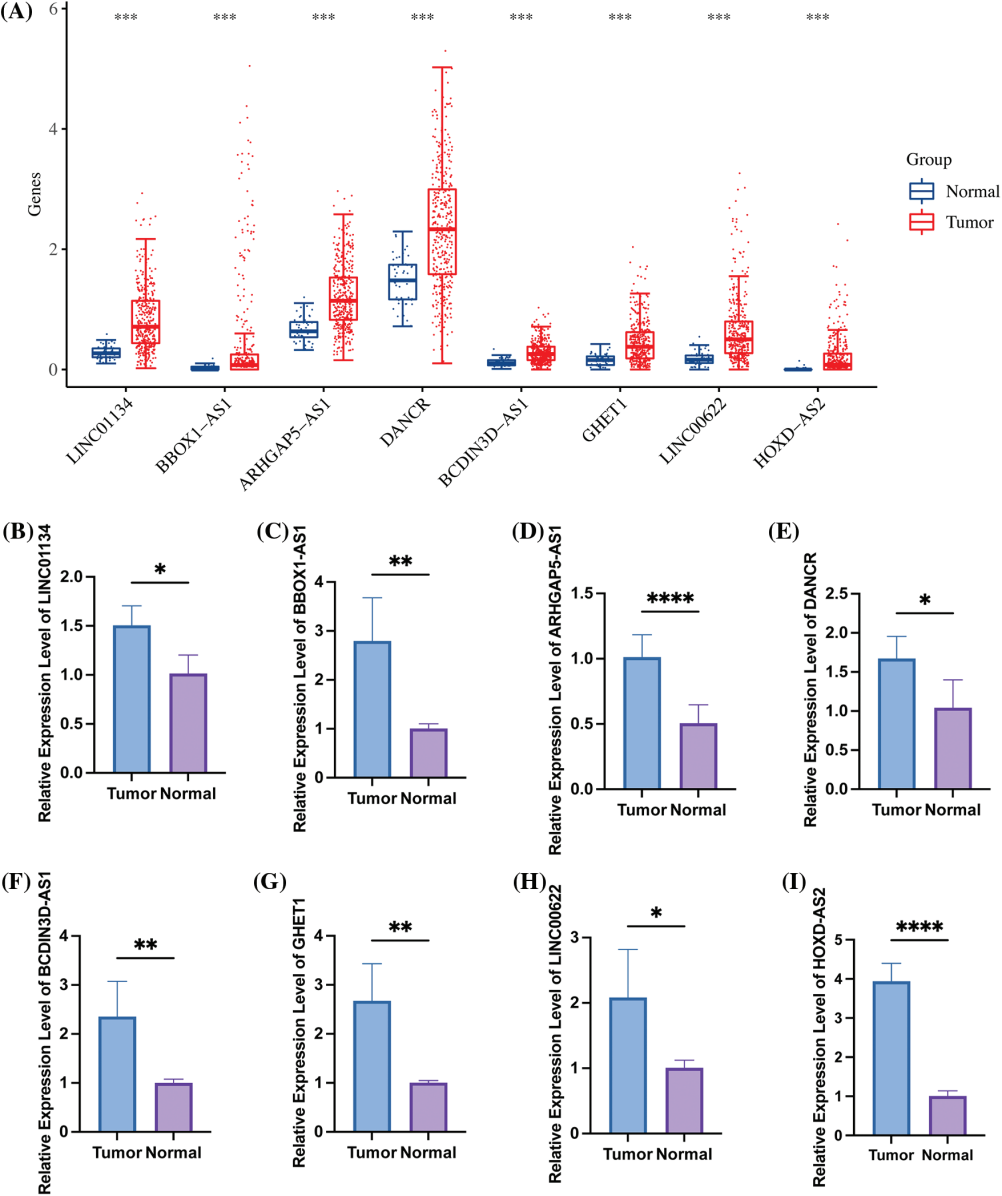
The high expression of 8 specific signatures in HCC. (A) Within the TCGA-LIHC dataset, all 8 signatures were found to be highly expressed in tumor samples. The figure further illustrates the expression levels of (B) LINC01134; (C) BBOX1-AS1; (D) ARHGAP5-AS1; (E) DANCR; (F) BCDIN3D-AS1; (G) GHET1; (H) LINC00622; and (I) HOXD-AS2 in both hepatocellular carcinoma cell lines and normal cell lines, as detected by qRT-PCR. Note: *p < 0.5; *p < 0.01; ***p < 0.001; ****p < 0.0001.

All eight identified signatures, namely LINC01134, BBOX1-AS1, ARHGAP5-AS1, DANCR, BCDIN3D-AS1, GHET1, LINC00622, and HOXD-AS2, exhibited high expression in HCC samples (Fig. 9A). When comparing hepatocellular carcinoma cell lines with normal cell lines, these signatures were found to be up-regulated (Figs. 9B–9I). This evidence leads to the suggestion that risk models involving these signatures might have substantial implications in the growth and evolution of HCC.

**Figure 10:**
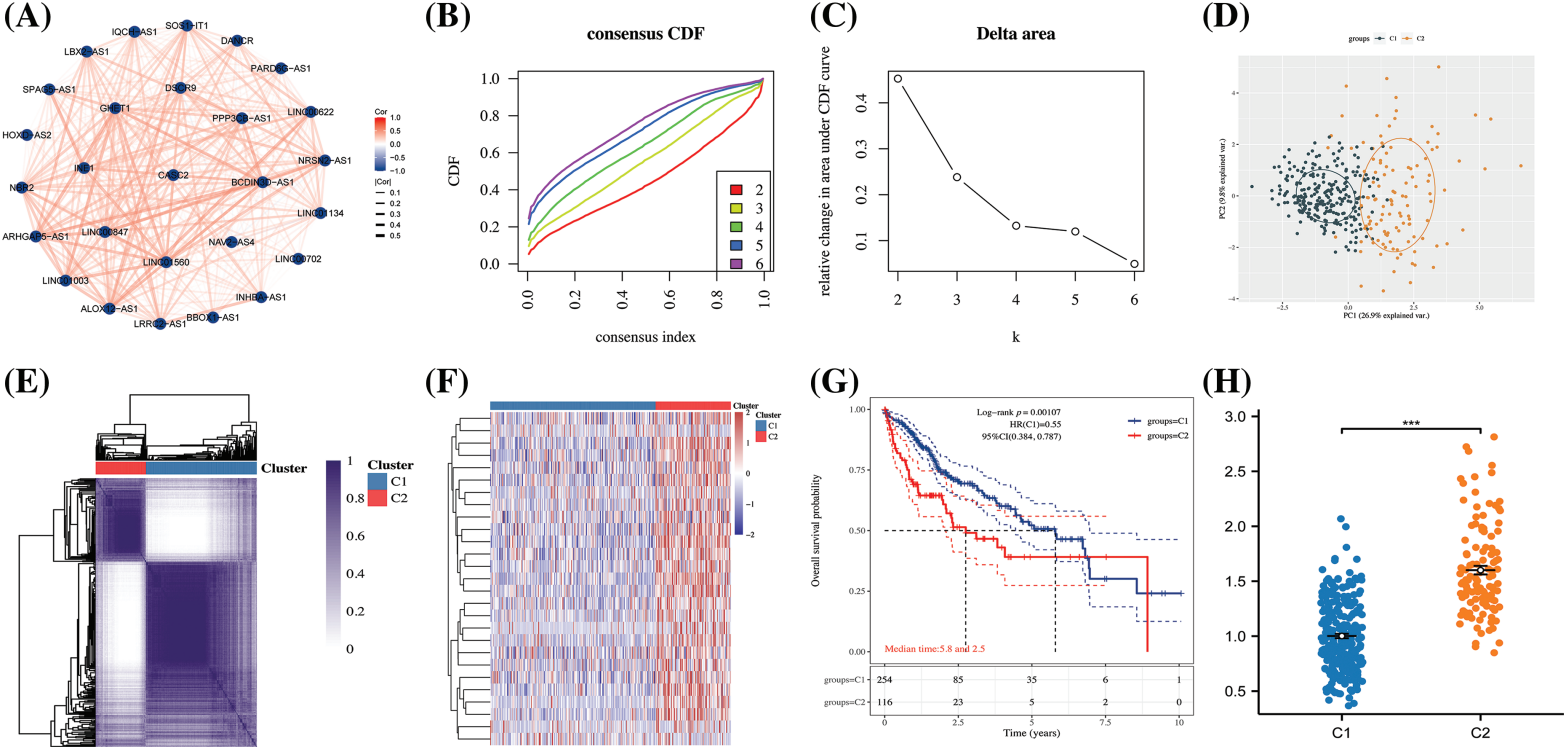
The construction of HCC subtypes based on ARLncRNAs. (A) A correlation network was created utilizing 27 ARLncRNAs; (B) The CDF (cumulative distribution function) curve, along with (C) the CDF Delta area curve, was employed through consensus clustering; (D) PCA (Principal Component Analysis) was conducted to assess the distribution of the two identified isoforms; (E) A heatmap was employed to represent the consensus matrix; (F) Another heatmap was utilized to distinctly illustrate the expression of the two subtypes; (G) The K-M curves depicted the survival patterns of the two subtypes; (H) The Riskscore analysis provided insight into the potential risks associated with the two subtypes. Note: ***p < 0.001.

To delve further into the potential role of ARLncRNAs in hepatocellular carcinoma (HCC), an analysis was conducted based on the expression profiles of 27 ARLncRNAs. We discovered correlations among them and subsequently clustered the expression profiles, determining that the optimal number of isoforms was two: C1 and C2 (Figs. 10B and 10C). These two isoforms could be distinctively differentiated (Figs. 10D–10F), and a notable finding was that the survival rate of patients in the C2 group was lower than that in the C1 group (Fig. 10G). Additionally, the Riskscore associated with the C2 group was found to be higher, emphasizing its significance in assessing potential risks (Fig. 10H).

**Figure 11:**
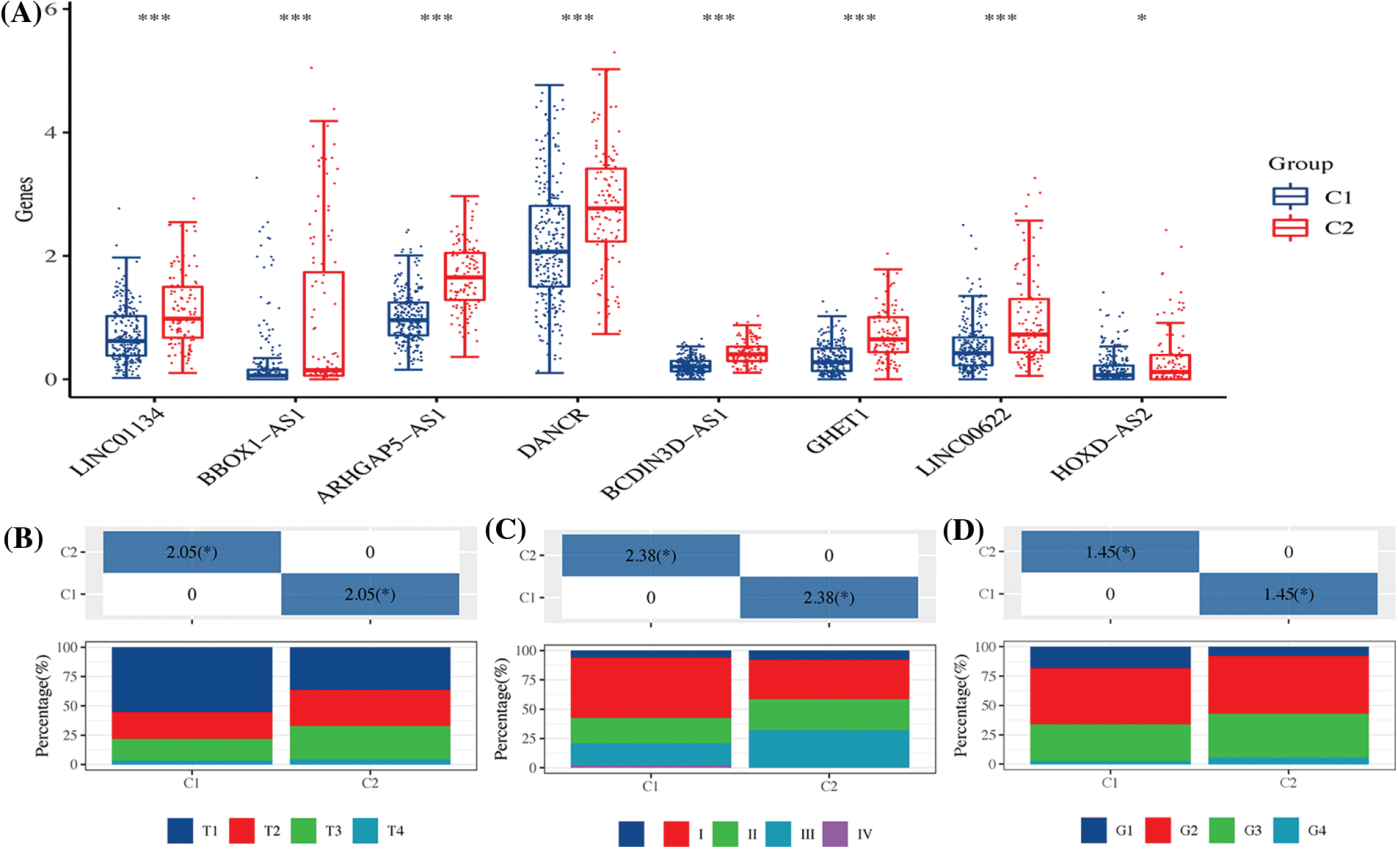
The expression of signatures and their clinical correlation across two HCC subtypes. (A) The figure showcases the expression of eight distinct signatures within the two HCC subtypes, highlighting the variations between them. It further details the differences in (B) T stage; (C) Overall stage; and (D) Grade between the two HCC subtypes, providing a comprehensive overview of their distinct characteristics. Note: *p < 0.5; ***p < 0.001.

These eight signatures demonstrated higher expression in the C2 group compared to the C1 group (Fig. 11A). Furthermore, the proportions of both T3+T4 (Fig. 11B) and Stage III&IV (Fig. 11C), along with Grade III&IV (Fig. 11D), were significantly more prevalent in patients of the C2 subtype. These statistics reveal a concerning trend: the C2 subtypes, characterized by high expression of ARLncRNAs, seemed to be associated with poorer clinical progression.

## Discussion

HCC is a frequently-seen malignant tumour worldwide [25]. Despite significant advancements in preventive, diagnosis and therapy of HCC, HCC is still the primary cause of death from cancer worldwide [26]. Because of the absence of effective prognostic biomarkers, HCC cases usually cannot get reasonable therapy immediately. In recent years, some scholars have questioned that the current TNM staging system cannot accurately forecast cancer patipatient’sgnosis, so it should be revised [27,28]. A growing number of studies have found that HCC has a variety of molecular characteristics and clinical results, which are strongly bound up with the prognosis of HCC patients according to exon sequencing, genomic characteristics and transcriptome analysis [29,30], autophagy takes different parts in different stages of HCC, which substantially affects the efficacy of various treatments.

Autophagy is one strongly transformed cellular process, which can maintain energy levels to circulate amino acids and other nutrients and renew cytoplasmic components [31]. It has dual functions with rregardto tumourigenesis: in normal cells, it takes a crucial part in monitoring damaged organelles, clearing aggregates, rand educing abnormal DNA and active oxidants to prevent somatic cells from changing into cancer cells [32]. Over the past few years, the research on the association of HCC with autophagy has been increasing [33]. For example, CD24 changes sorafenib resistabyough activating autophagy in HCC [34]. CHD1L enhances autophagy-mediated migration of HCC via ZKSCAN3 [35]. In recent years, as high-throughput sequencing develops, several bioinformatics studies have been conducted on various types of cancer to determine useful indexes of prognosis/therapeutic targets [36–38], such as methylation biomarkers, whole genome predictors, and prognostic lncRNA [39,40]. Especially, since the discovery that lncRNA takes a crucial part in many cellular processes, research on lncRNA is now quite active. However, there are few reports of autophagy-associated lncRNAs in HCC, and there is little detailed and full analysis of the relationship of lncRNA expression with autophagy in HCC’ nosis.

In the present study, 27 ARLnRNAs were identified by analyzing lncRNAs from 371 tumor samples within the TCGA database. Through univariate Cox analysis, 13 ARLnRNAs were found to be associated with HCC prognosis. The results highlighted that the eight carefully screened ARLnRNA signatures were notably correlated with the prognosis of HCC patients. The validity of this risk model was confirmed from various angles and using multiple approaches, demonstrating that patients in the high-risk group had a less favorable prognosis and survival rate. Distinct differences in immune function were observed among different risk levels, possibly linked to tumor progression. Enrichment analyses indicated associations with metabolism and cell cycle mitophagy. Chao et al. [41] observed in their research that autophagy, a highly conserved metabolic process in HCC, aids the progression of existing liver tumors. Tumor cells in hepatocellular carcinoma, when stimulated by stressors like hypoxia, vigorously activate autophagy, thus promoting tumor growth by supplying nutrients and minimizing anti-tumor immune responses [42–44]. Yang et al. [45] identified seven autophagy-associated lncRNAs as potential prognostic and therapeutic targets for HCC, which aligns with our findings. Our study, however, provided a more detailed analysis of a risk model comprising ARLncRNAs for the prognostic assessment of HCC. We employed multiple algorithms and datasets to evaluate the model’s validity and validated its applicability across a vast majority of populations with diverse clinical characteristics. We further compared differences in various immune scores and Stem Scores, providing substantial data to support our results.

Furthermore, the current study uncovered that Paclitaxel appears to be an ideal therapeutic agent for treating individuals at high risk of HCC. Given the extensive research on the role of autophagy in various cancers, Paclitaxel has shown effectiveness as a conventional anticancer drug [46]. However, a significant challenge lies in the fact that Paclitaxel-induced autophagy may lead to resistance to the drug’s own anticancer properties [46,47]. Recent research has shed light on potential solutions, indicating that the inhibition of autophagic processes in tumor cells can restore sensitivity to Paclitaxel, and that encapsulating Paclitaxel with nanoparticles or liposomes may enhance its anticancer effects [48–50]. These insights align with the findings of our study, where resistance to Paclitaxel is more marked in the high-risk HCC population. If the effectiveness of Paclitaxel can be augmented for patients with a more dismal prognosis, it may pave the way for more precisely tailored treatments for individuals at different stages of HCC.

Moreover, we successfully confirmed the elevated expression of LINC01134, BBOX1-AS1, ARHGAP5-AS1, DANCR, BCDIN3D-AS1, GHET1, LINC00622, and HOXD-AS2 through *in vitro* experiments. The high expression of these signatures could potentially identify a specific subtype for HCC patients, offering insights into disease progression and enhancing prognostic risk assessments to prompt alerts. Nevertheless, this study is not without limitations. For instance, we did not analyze the clinical samples that were collected. Additionally, a lack of comprehensive clinical data, particularly related to T-stage and Stage and Grade-stage HCC, hindered our ability to definitively determine the risk level within our cohort for the time being. Lastly, further validation through wet-lab experiments is needed to understand the potential functionality of the model. As we move forward, we plan to conduct additional tests and research to reinforce our findings.

## Conclusions

In conclusion, this study led to the development of a risk model comprised of eight ARLncRNAs, extracted from a specific database. The Riskscore generated by this model serves as an independent factor in predicting OS in patients with HCC. As a result, it offers a valuable tool for evaluating the prognosis of HCC.

## Supplementary Materials





## Data Availability

All data from this study can be obtained by contacting the corresponding author.

## Supplementary Materials

The supplementary material is available online at https://doi.org/10.32604/or.2023.030988.
