# Supplementary material for: Developing risk models and subtypes of autophagy-associated LncRNAs for enhanced prognostic prediction and precision in therapeutic approaches for liver cancer patients
Source: Oncol Res. 2024 Mar 20;32(4):703–16. doi: 10.32604/or.2023.030988 (PMC10972734; doi:10.32604/or.2023.030988)
Supplement: Supplementary file 1 [file OncolRes-32-30988-s001.docx]

Supplementary Table 1 Primer sequences

|  | | Sequence (5'->3') | Length | Start | Stop | Tm | GC% |
| --- | --- | --- | --- | --- | --- | --- | --- |
| LINC01134 | Forward primer | GCCACCCTGGACTCCTAAAC | 20 | 1311 | 1330 | 60.04 | 60 |
|  | Reverse primer | GCGGACAGAAATCCCCTTGA | 20 | 1433 | 1414 | 60.04 | 55 |
| BBOX1-AS1 | Forward primer | AATACCAAAGAGGGCCGCTG | 20 | 440 | 459 | 60.39 | 55 |
|  | Reverse primer | TAGGGAGTGACTGGGGTCAG | 20 | 914 | 895 | 59.96 | 60 |
| ARHGAP5-AS1 | Forward primer | GATCGCTCGCCAACTACAGA | 20 | 430 | 449 | 59.9 | 55 |
|  | Reverse primer | GACCTCGGGAAAGTCACAGG | 20 | 903 | 884 | 60.04 | 60 |
| DANCR | Forward primer | AATGCAGCTGACCCTTACCC | 20 | 585 | 604 | 60.03 | 55 |
|  | Reverse primer | GGCTTCGGTGTAGCAAGTCT | 20 | 681 | 662 | 60.04 | 55 |
| BCDIN3D-AS1 | Forward primer | GGACCTCCCTTCCCCCTTAT | 20 | 66 | 85 | 60.03 | 60 |
|  | Reverse primer | CAGCCAGAGGTAAGTGGCAT | 20 | 1051 | 1032 | 59.75 | 55 |
| GHET1 | Forward primer | GCCCCAAGCCAATGGAAAAG | 20 | 694 | 713 | 60.04 | 55 |
|  | Reverse primer | CAGTGCATGCAAAGGCAGAG | 20 | 1111 | 1092 | 60.11 | 55 |
| LINC00622 | Forward primer | GCCCTCCTCCCATGTTGAAA | 20 | 232 | 251 | 59.96 | 55 |
|  | Reverse primer | GCAGGCAAAGGTGTGTGATG | 20 | 430 | 411 | 60.04 | 55 |
| HOXD-AS2 | Forward primer | GCTGTAATCCACTAGGCGCT | 20 | 143 | 162 | 59.89 | 55 |
|  | Reverse primer | TATGCCCATGAGGGGAATGC | 20 | 550 | 531 | 59.89 | 55 |
| GAPDH | Forward primer | AATGCAGCTGACCCTTACCC | 20 | 585 | 604 | 60.03 | 55 |
|  | Reverse primer | GGCTTCGGTGTAGCAAGTCT | 20 | 681 | 662 | 60.04 | 55 |
