# Supplementary material for: Developing risk models and subtypes of autophagy-associated LncRNAs for enhanced prognostic prediction and precision in therapeutic approaches for liver cancer patients
Source: Oncol Res. 2024 Mar 20;32(4):703–16. doi: 10.32604/or.2023.030988 (PMC10972734; doi:10.32604/or.2023.030988)
Supplement: Supplementary file 2 [file OncolRes-32-30988-s002.docx]

Supplementary Table 2 LncRNAs associated with autophagy

| **Symbol** | **Description** | **Symbol** | **Description** |
| --- | --- | --- | --- |
| ALOX12-AS1 | ALOX12 Antisense RNA 1 | LINC00702 | Long Intergenic Non-Protein Coding RNA 702 |
| ARHGAP5-AS1 | ARHGAP5 Antisense RNA 1 (Head To Head) | LINC00847 | Long Intergenic Non-Protein Coding RNA 847 |
| BBOX1-AS1 | BBOX1 Antisense RNA 1 | LINC01003 | Long Intergenic Non-Protein Coding RNA 1003 |
| BCDIN3D-AS1 | BCDIN3D Antisense RNA 1 | LINC01134 | Long Intergenic Non-Protein Coding RNA 1134 |
| CASC2 | Cancer Susceptibility 2 | LINC01560 | Long Intergenic Non-Protein Coding RNA 1560 |
| DANCR | Differentiation Antagonizing Non-Protein Coding RNA | LRRC2-AS1 | LRRC2 Antisense RNA 1 |
| DSCR9 | Down Syndrome Critical Region 9 | NAV2-AS4 | NAV2 Antisense RNA 4 |
| GHET1 | Gastric Carcinoma Proliferation Enhancing Transcript 1 | NBR2 | Neighbor Of BRCA1 LncRNA 2 |
| HOXD-AS2 | HOXD Cluster Antisense RNA 2 | NRSN2-AS1 | NRSN2 Antisense RNA 1 |
| INE1 | Inactivation Escape 1 | PARD6G-AS1 | PARD6G Antisense RNA 1 |
| INHBA-AS1 | INHBA Antisense RNA 1 | PPP3CB-AS1 | PPP3CB Antisense RNA 1 (Head To Head) |
| IQCH-AS1 | IQCH Antisense RNA 1 | SOS1-IT1 | SOS1 Intronic Transcript 1 |
| LBX2-AS1 | LBX2 Antisense RNA 1 | SPAG5-AS1 | SPAG5 Antisense RNA 1 |
| LINC00622 | Long Intergenic Non-Protein Coding RNA 622 |  |  |
